# A database of circadian and diel rhythmic gene expression in the yellow fever mosquito *Aedes aegypti*

**DOI:** 10.1186/1471-2164-15-1128

**Published:** 2014-12-17

**Authors:** Matthew T Leming, Samuel SC Rund, Susanta K Behura, Giles E Duffield, Joseph E O’Tousa

**Affiliations:** Department of Biological Sciences and Eck Institute for Global Health, University of Notre Dame, Galvin Life Sciences Bldg, Notre Dame, IN 46556 USA; Center for Immunity, Infection and Evolution, School of Biological Sciences, University of Edinburgh, Edinburgh, EH9 3JT UK

**Keywords:** *Aedes aegypti*, Circadian rhythm, Database, Diel rhythm, Microarray, Gene expression

## Abstract

**Background:**

The mosquito species *Aedes aegypti* is the primary vector of many arboviral diseases, including dengue and yellow fevers, that are responsible for a large worldwide health burden. The biological rhythms of mosquitoes regulate many of the physiological processes and behaviors that influence the transmission of these diseases. For insight into the molecular basis of biological rhythms, diel and circadian gene expression profiling has been carried out for many species. To bring these resources to *Aedes aegypti* researchers, we used microarray technology to carry out a genome wide assessment of gene expression during the 24 hour light/dark (LD) cycle and during constant darkness (DD). The purpose of this report is to describe the methods, the validation of the results, and the organization of this database resource.

**Description:**

The *Aedes aegypti* Circadian Database is a publicly accessible database that can be searched via a text-based query to visualize 44 hour temporal expression patterns of a given gene in *Ae. aegypti* heads under diel (observed under a 12 hour/12 hour LD cycle) and circadian (observed under DD) conditions. Profiles of gene expression under these conditions were assayed by Nimblegen 12-plex microarrays and rhythmicity was objectively assessed by the JTK_CYCLE algorithm. The output of the search is a graphical representation of the expression data along with computed period length, the time-of-day of gene expression peaks, and statistical determination for rhythmicity.

**Conclusion:**

Our results show that at least 7.9% of the gene set present in the *Aedes aegypti* head are rhythmic under LD conditions and 6.7% can be considered circadian, oscillating under constant dark conditions. We present these results in the *Aedes aegypti* Circadian Database through Bioclock, a public website hosted by the University of Notre Dame at http://www.nd.edu/~bioclock/. This website allows searchable browsing of this quantitative gene expression information. The visualization allows for gene-by-gene comparison of transcript expression under both diel and circadian conditions, and the results are presented graphically in a plot profile of gene expression. The *Ae. aegypti* Circadian Database provides a community resource for observing diel and circadian fluctuations in gene expression across the *Ae. aegypti* genome.

## Background

Many vector borne diseases including yellow fever, dengue fever, Japanese encephalitis and Chikungunya fever are transmitted by the mosquito species *Aedes aegypt*i. These infections not only cause morbidity and further economic burden in predominantly low-income countries, but also result in a substantial death toll worldwide [1–3]. Sensory systems in these mosquitoes have been a focus of study for many years in the hopes of improving intervention strategies. Recently, investigations into the circadian rhythms of other insect species, including mosquitoes, have demonstrated its interaction with key sensory systems [4, 5].

The circadian clock regulates RNA expression eventually influencing behavioral output. The circadian clock is an endogenous mechanism comprised of a series of transcriptional-translational feedback loops (TTFLs), that can be entrained by external stimuli (in particular light), and that drives the rhythmic expression of genes [6, 7]. In the mosquito model, much of which is inferred from model insect species, the proteins period (PER), timeless (TIM) and CRY2 form the negative TTFL negative loop, and clock (CLK) and cycle (CYC) the positive TTFL loop [5, 8–14]. The clock is reset/entrained by light, and the insect cryptochrome 1 (CRY1) functions as a photoreceptor in this process, while CRY2 contributes to the negative TTFL of the circadian clock [10–12]. Output from the clock is through the generation of rhythms at the transcriptional, post-transcriptional and post-translational regulation levels, and that ultimately result in biochemical and physiological rhythmicity [8, 15, 16]. Previous studies have demonstrated that ~2-10% of the transcriptome is under rhythmic regulation, depending upon species, tissue, strain and specific investigation [8, 15, 17–19]. The circadian clock in the mosquito species *Anopheles gambiae* regulates the expression of many RNA transcripts as well as behaviors, including flight and feeding [8, 20]. Furthermore, daily rhythms in gene expression are implicated in driving physiological changes in *An. gambiae*, with time-of-day specific changes in odorant sensitivity, biting behavior, metabolic detoxification and in resistance/susceptibility to pesticides [20, 21]. Behaviors occurring with a daily rhythm in *Ae. aegypti* include biting, flight, oviposition, and sugar feeding [22–26]. Furthermore, the circadian clock is implicated in the control of physiological processes in *Ae. aegypti* ranging from permethrin insecticide resistance to rhodopsin management within the visual system [27, 28]. Characterizing the impact of the circadian clock on these and other physiological processes is crucial to understanding how these behaviors are regulated.

A genome-wide study of 24 hour rhythmicity in *Ae. aegypti* in mosquito heads collected under light/dark (LD) cycle conditions has been conducted previously on a mixed laboratory strain population derived from Chapas, Mexico [9]. An additional study used these data in a meta-analysis, to compare *Ae. aegypti* and *An. gambiae* rhythmic gene expression [19]. Our current work expands on these studies by providing information on rhythmicity in *Ae. aegypti* heads under both LD cycle and constant dark (DD) conditions, allowing analysis of gene rhythmicity driven by the endogenous clock or rhythmicity regulated by light and the LD cycle. In fact in *An. gambiae* mosquitoes and *Drosophila* there is evidence for a dual control mechanism regulating 24 hour rhythmicity, involving both the entrainable endogenous clock and direct LD cycle influences shaping rhythmicity [8, 18, 19]. Our database provides information on *Ae. aegypti* gene expression under both diel (observed under a LD cycle) and circadian (observed under DD) conditions. The *Aedes aegypti* Circadian Database allows for easy access to information on *Ae. aegypti* gene expression.

## Construction and content

This study consisted of collection and analysis of mosquito samples, exposed to either a 12 hour light/12 hour dark cycle or maintained in constant darkness, at every four hours through a two day period. RNA extracted from heads of these mosquito samples were analyzed by Nimblegen microarrays. qPCR analysis was performed on a set of canonical circadian clock genes to validate our experimental method. We then created the *Ae. aegypti* Circadian Database, hosted at the Bioclock website at the University of Notre Dame [8], for use by the scientific community. The database allows queries into daily profiles of individual *Ae. aegypti* genes. Objective analysis of rhythmicity was computed by the JTK_CYCLE algorithm [29]. The expression profile of each gene can also be visually inspected in a graphical format at this site. Further details of these experimental protocols are described below.

### Sample collection

For consistent management of the mosquito samples, 24 cages were prepared 36 hours prior to the start of tissue collections, and ~30 presumed mostly mated non-blood fed female *Ae. aegypti* Higgs White eye (Wh) strain mosquitoes [30], aged 2–6 days, were randomly assigned to each cage, thereby containing mixed aged animals. Mosquitoes were placed in separate light-tight boxes with controllable lighting systems as carried out previously for *An. gambiae* mosquitoes [31]. The light/dark (LD)-reared mosquitoes were maintained on a 12 hour/12 hour LD cycle (11 hour full light [~110 lux], 11 hour darkness, and 1 hour dawn and 1 hour dusk transitions). The dawn transition consisted of a one hour linear increase from darkness to approximately 20% of the daylight level followed immediately by an increase to full daylight levels. In a reciprocal manner, the dusk period started with an immediate decline to approximately 20% of full daylight intensity followed by the one hour linear reduction to complete darkness. DD mosquitoes were maintained in constant darkness during the experiment. All mosquitoes were provided with 5% high fructose corn syrup *ad libitum* [8, 31]. Collections of mosquito samples were identified under LD conditions in terms of Zeitgeber time (ZT) where ZT0 is the onset of dawn and ZT12 is the onset of the dusk cycle; and under DD conditions in terms of circadian time (CT) with CT0 defined as the onset of subjective dawn inferred from the previous LD cycle. To acclimate the mosquitoes to their new environment, boxes for both LD and DD groups were kept under normal LD conditions until the dusk before ZT/CT0 of day 1 of collections. One cage from each LD and DD box was collected every four hours for 44 hours. Mosquitoes, aged 4–9 days, were euthanized on dry ice and stored at -80°C. Heads were removed from bodies on an ice chilled metal plate while frozen and collected. Care was taken to ensure the intact head included all sensory appendages. The RNeasy RNA Isolation Kit (Qiagen) was used to isolate RNA from each sample, following manufacturer’s instructions except the final step where genomic DNA was removed by using the DNA-free Kit (Applied Biosystems) per manufacturer instructions.

### RNA preparation and hybridization

Analysis of RNA and microarray hybridizations were performed in the University of Notre Dame Genomics & Bioinformatics Core Facility. A Nanodrop ND-2000 (ThermoScientific) was used to determine total RNA and double stranded cDNA concentrations. A Bioanalyzer 2100 (Agilent Technologies) and RNA 6000 Nano kit (Agilent Technologies) were used to test for the integrity/degradation of RNA based on the presence of distinct ribosomal RNA peaks. Only high quality, not degraded, RNA samples were used. RNA was then converted to double stranded cDNA using the TransPlex Complete Whole Transcriptome Amplification Kit (Sigma Aldrich). RNA degradation, double stranded cDNA purification, and cDNA precipitation were conducted per NimbleGen Gene Expression Array user's guide. Labeling double stranded cDNA was performed using NimbleGen Single Color Labeling Kit (Roche NimbleGen).

### Microarray analysis

Hybridization and post-hybridization wash were carried out using the Hybridization Kit, LS (Roche NimbleGen) and Wash Buffer Kit (Roche NimbleGen). Labeled cDNA samples were randomized and hybridized on custom designed Nimblegen 12-Plex microarray chips, designed at the University of Notre Dame [GEO accession# GPL18530]. This chip design includes 15,202 genes representing >80% of the 18,838 genes identified in *Ae. aegypti* (AaelL1.1; VectorBase.org). The 12-plex Nimblegen platform was selected to make use of newer microarray technology. The benefits include global gene expression profiling based on a genome-wide oligonucleotide high definition array [32]. Multiple probes for the entire standard gene set of *Ae. aegypti* genes are present on these arrays. Also the Nimblegen platform generates these probes using photolithography with masks technology instead of the inkjet method for fabrication employed by the Agilent microarrays. Ptitsyn *et al*. (2011) previously used the Agilent cDNA microarray platform to evaluate rhythmic expression in the *Ae. aegypti* genome [9]. In contrast, a genome-wide oligo microarray was used in the current study. This was chosen to enhance comprehensive representation of probes of the official gene set of *Ae. aegypti*, increase homogeneity of the spots in the array, and to perform sample hybridizations in a highly multiplexed format.

The image acquisition was performed using a NimbleGen MS 200 Microarray Scanner at 2 μM resolution. Array JPEGs were aligned with a grid and processed using the RMA (Robust Multi-array Average) algorithm [33] to determine relative probe intensity using Version 2.5 of the NimbleScan software. The RMA algorithm has proven to be a reliable data normalization method for microarrays [34]. This algorithm adjusts for background noise using raw intensity data. The log_2_ transformed value of each background corrected perfect match probe is obtained and used for quantile normalization. The multi-array analysis is then carried out on the quantiles to perform robust multi-array average calculations.

As a result of the chip design, normalization with the RMA algorithm returned expression values per gene. Utilizing all probes per gene resulted in a higher threshold for determination of rhythmicity for a single gene within our database. However, the raw individual probe values are available for additional computational analysis at the National Center for Biotechnology Information Gene Expression Omnibus (NCBI-GEO, GEO accession# GSE60496) and at VectorBase.org.

Data from each chip were collated with respective time points into a LD and DD file respectively. The RMA normalized data were analyzed using the JTK_CYCLE algorithm [29], a statistical method for detecting oscillations in gene expression by using the Jonckheere-Terpstra-Kendall (JT) test and the Kendall’s Tau (rank correlation) (K). The *p*-value defined by JTK_CYCLE is a determination of likelihood for an individual gene to be rhythmic in a defined period length, in this case 20–28 hour in LD and 18–28 hour in DD. Any gene with a *p*-value that scored <0.05 was considered to be rhythmic. The output of JTK_CYCLE also provided a false discovery rate (FDR) *q*-value for each gene that is also accessible via Bioclock. However, it should be noted that the derived *q*-values may not represent accurate rate of false positives from our data due to the single-replicate time course experimental design of our study.

JTK_CYCLE was used to search for rhythmic genes within a pre-defined period length range. The period length, 24.0 hours under entrained conditions, is known in mosquitoes to shorten under constant dark conditions: *An. gambiae* has a free running period of ~23 hours [8, 31], and in *Ae. aegypti* (Rockefeller strain) the endogenous period length was ~22 hours in adult females as determined from locomotor/flight activity profiles collected in DD [14]. Similar results were seen in the Wh strain female mosquitoes used in this study, exhibiting a shortened free-running period length of 22.88 +/- 0.14 hours (mean +/- SEM) in flight/locomotor activity assays (unpublished data). To accommodate this predicted and evident shorter free running period we searched genes with period lengths of 20–28 hours in LD and expanded the window to 18–28 hours for the data collected in DD. In LD, the mean period length of rhythmic genes was observed at 24.8 hours. DD rhythmic genes however showed a shorter mean period length of 23.4 hours, representing a 1.4 hour shortening of the average period length from LD. Note that in our subsequent analysis only a single gene was identified as rhythmic with a period <20 hour.

To determine the background level of fluorescence, using the Cluster 3.0 program, the expression data was log_2_ transformed, mean centered, and normalized across the time course for each gene. We then identified a group of genes that showed minimal expression across the experimental samples [35]. The Pearson correlation was used as a distance measure to cluster the expression variation for identification of different gene groups as previously described [36, 37]. From this we identified a group of 1,161 genes that were expressed significantly lower than other genes in *Ae. aegypti* heads. The expression levels of these genes were used to determine background fluorescence values of the microarray data (LD, 52.8; DD, 51.7). Any gene with an average expression value below the cutoff was considered as background and not used for calculation of the total number of rhythmic genes.

**Figure 1:**
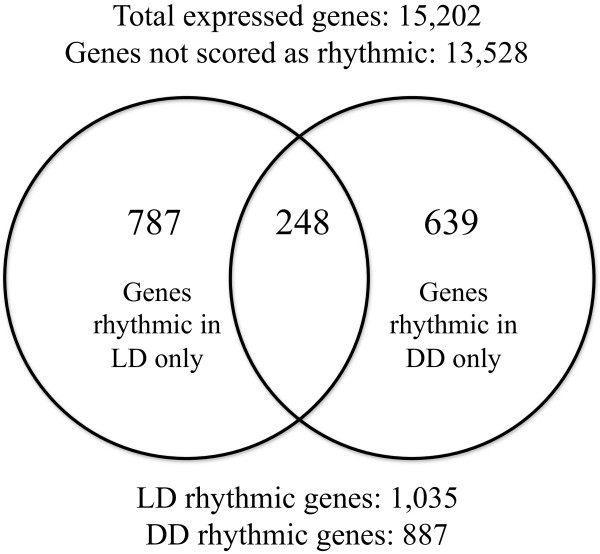
**Venn diagram showing the number of rhythmic genes observed under LD and DD conditions.** The Venn diagram depicts different classes of genes in the mosquito head, including rhythmic only under LD conditions, rhythmic only under DD conditions, rhythmic under both LD and DD conditions, and genes not scored as rhythmic.

**Figure 2:**
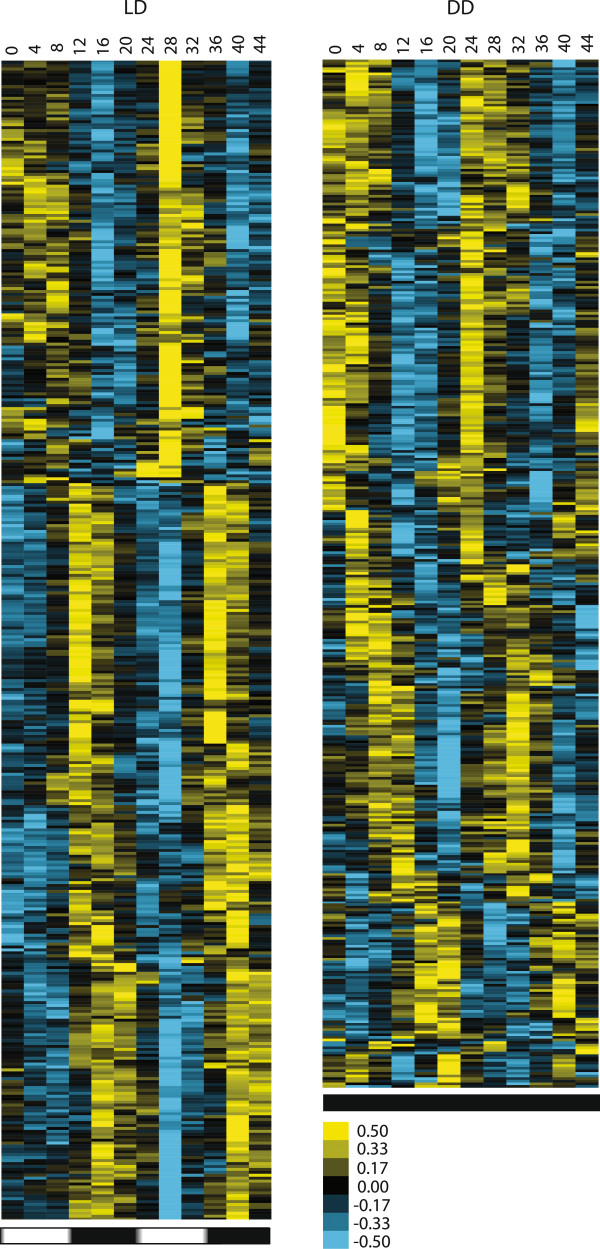
**Heatmap of genes found to be rhythmic under LD (left) and DD (right) conditions.** Images show the hierarchical clustering of genes found to be rhythmic. The time points taken during the 44 hour experimental period are shown above the images, and horizontal black/white or black bars below the images indicate the day/night or constant dark conditions. An expression intensity key is displayed in lower right. Yellow represents higher expression, and blue represents lower expression, relative to the mean expression value for each gene.

In summary, a gene was determined to be rhythmic if it (*i*) scored a *p*-value < 0.05 as determined by the JTK algorithm; (*ii*) had a period length between 20–28 hours in LD or 18–28 hours in DD; and (*iii*) had a mean fluorescence intensity across all time points of >52.8 and >51.7, under LD and DD conditions, respectively. After these criteria were applied there were a total of 1,035 rhythmic (LD) and 887 circadian (DD) genes that were identified. Specific categories of gene rhythmicity are depicted in a Venn diagram (Figure 1). Two interesting findings emerge from these results: firstly, the numbers of genes rhythmic in LD conditions is greater than under DD (787 versus 693 genes respectively); and secondly, there is limited overlap of genes rhythmic under both conditions (24% and 28% as proportions of LD or DD rhythmic genes). Such limited overlap has been observed before in circadian microarray studies of *An. gambiae* mosquitoes and *Drosophila* [7, 8, 15, 18], as has the occurrence of fewer genes rhythmic under DD conditions [8, 15, 17, 18]. These findings are consistent with there being a direct effect of light on regulating gene expression. Profiles of all rhythmic genes are displayed in separate LD and DD condition heatmaps (Figure 2). The centroid linkages generated from the Cluster 3.0 analysis were visualized as heatmaps using Java TreeView [37, 38].

### Validation of microarray analysis

**Figure 3:**
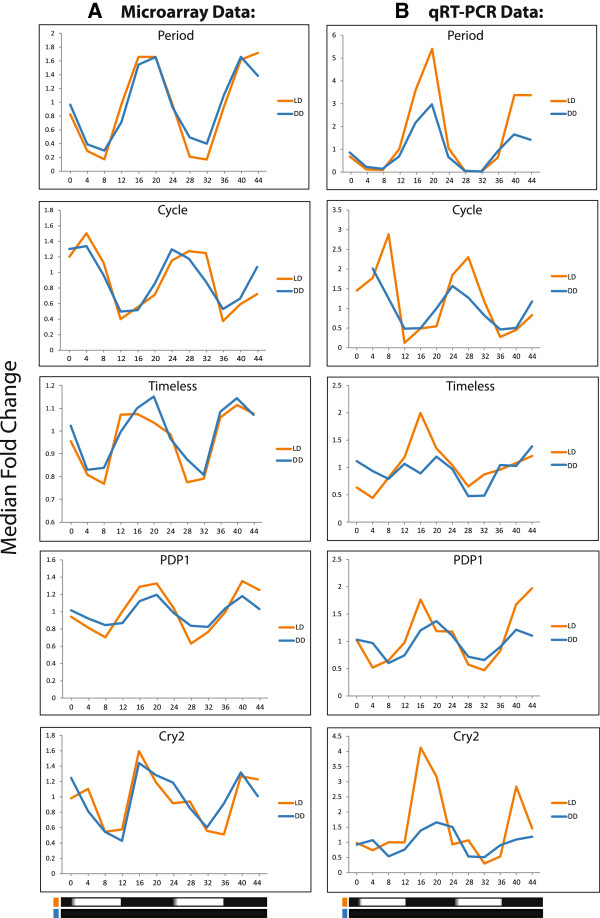
**Expression profiles of clock genes determined by qPCR verify microarray analysis.** Individual profiles of microarray data demonstrate diel and circadian profiles of a set of clock genes **(A)**. These profiles were then verified by qRT-PCR **(B)**. *Cycle* gene (*aeCYC*, *circadian protein clock/arnt/bmal/pas*, AAEL002049) is the ortholog of *An. gambiae cycle* (*CYC*, AGAP012873) and *Drosophila cycle* (*cyc*); and *cryptochrome 2* (*aeCRY2*, *DNA photolyase*, AAEL002602) is the ortholog of *An. gambiae cryptochrome 2* (*CRY2*, AGAP004261). Values are normalized by median fold change across the time course. Description of genes and primers used for qPCR are in Table 1. Horizontal black/white or black bars represent the day/night or constant dark collection conditions.

To validate the expression profiles generated from the microarrays, qRT-PCR analysis using SYBR Select (Invitrogen) was carried out using the 7500 Fast Real-Time PCR System (Applied Biosystems). Primers were designed using the NCBI primer design tool to produce a product size of 50–150 bases and spanned consecutive exon pairs. Specificity was confirmed by using the Basic Local Alignment Search Tool (BLAST) hosted by NCBI. Using the same cDNA used for the microarray experiments we screened a set of genes known to function in the insect circadian clock (Table 1). For any genes not named on VectorBase.org we relied on orthologous sequences in *An. gambiae* and *Drosophila*. We calculated the ΔΔCT of expression profiles normalized against the *40s ribosomal protein s17* (*aeRps17*, AAEL004175), previously shown to be appropriate for normalization in *Ae. aegypti* [39] and also showed no rhythmicity under LD or DD conditions in our current data. Resulting profiles were converted to median fold change and compared with profiles generated from the microarray dataset (Figure 3). Times of peak expression for this set of clock genes are in agreement with those previously reported in *Ae. aegypti*, where peak expression of *PER* and *TIM* is at ZT/CT18-19, and *CLK* and *aeCYC* is at ZT/CT2-4 [13, 14, 29]. These data confirm that our experimental design and execution is capable of detecting the rhythmic expression of individual genes.

**Table 1:** **Genes used for qRT-PCR verification of gene expression rhythmicity**

Gene:	GeneID:	qPCR primer sequence:
*timeless* (*TIM*)	AAEL006411	F - GGCTTTCTCGTACAAGAGCTTC
		R - GAACTTCAGGAAATAGGTCACCA
*cycle* (*aeCYC*)	AAEL002049	F - GAGCAGTTGTCTTCTTCGGATT
		R - CACAGCCTTGTCACACCTTG
*period* (*PER*)	AAEL008141	F - GGAGCAATTCGTCCGGTAG
		R - TGGGTTCAACAGACGTTTTC
*PAR-domain protein 1* (*PDP1*)	AAEL005255	F - GACGCATGAAGGAGAATCAA
		R - TCCATGTTTTCCTGTTTCAGC
*DNA photolyase* (ae*CRY2*)	AAEL002602	F - GCAACTACGCCACAGTACGA
		R - GCTTCCGTTTCACCTCTCAC
*40S ribosomal protein S17* (ae*RPS17*)	AAEL004175	F - AGACAACTACGTGCCGGAAG
		R - TTGGTGACCTGGACAACGATG

The *Ae. aegypti* gene name, GeneID and sequence of forward and reverse primers are given. The 40s ribosomal protein S17 was used as a non-rhythmic standard to calculate ΔΔCT.

## Utility and discussion

Our goal was to generate a publicly available database that allows for visualization of the temporal expression patterns of *Ae. aegypti* genes under LD and DD conditions. By accessing this information, investigators can quickly and conveniently determine the ~24 hour rhythmicity and circadian phase (time of peak expression) of any gene of interest contained within the dataset. Only three other databases of this kind have been developed: one for circadian studies of mice and *Drosophila* (http://expression.gnf.org/circadian) that was in existence for over 10 years [17, 40], and which was superseded by the Circa database (http://bioinf.itmat.upenn.edu/circa) [41], and the original Bioclock database developed for *An. gambiae* mosquitoes [8]. In the case of the *An. gambiae* data, there is evidence for the cooperative influence of both the light/dark cycle and the circadian clock in regulating rhythmic gene expression [8, 19]. The addition of the *Ae. aegypti* Circadian Database extends our understanding of the circadian and diel regulation of 24 hour rhythmicity, including comparisons made between species of mosquitoes and other dipterans. Diel rhythms were compared between *An. gambiae* and *Ae. aegypti* previously and demonstrate similarities in rhythmic expression in several processes, including the vesicular-type ATPase (V-ATPase) and its subunits, and the visual system [19]. Earlier studies suggested that V-ATPase genes have roles in *Ae. aegypti* susceptibility to dengue virus infection [42] indicating that rates of disease transmission may be influenced by diel rhythms. The regulation of these systems and other influences of the circadian clock can now be investigated by use of the *Ae. aegypti* Circadian Database. The database is currently limited to the expression profiles for the white-eyed Wh strain of *Aedes aegypti*. The database establishes a foundation to explore gene expression differences that might exist among this strain, other established laboratory mosquito strains, and more recent collections of mosquitoes such used in the Ptitsyn et al. [9] study.

**Figure 4:**
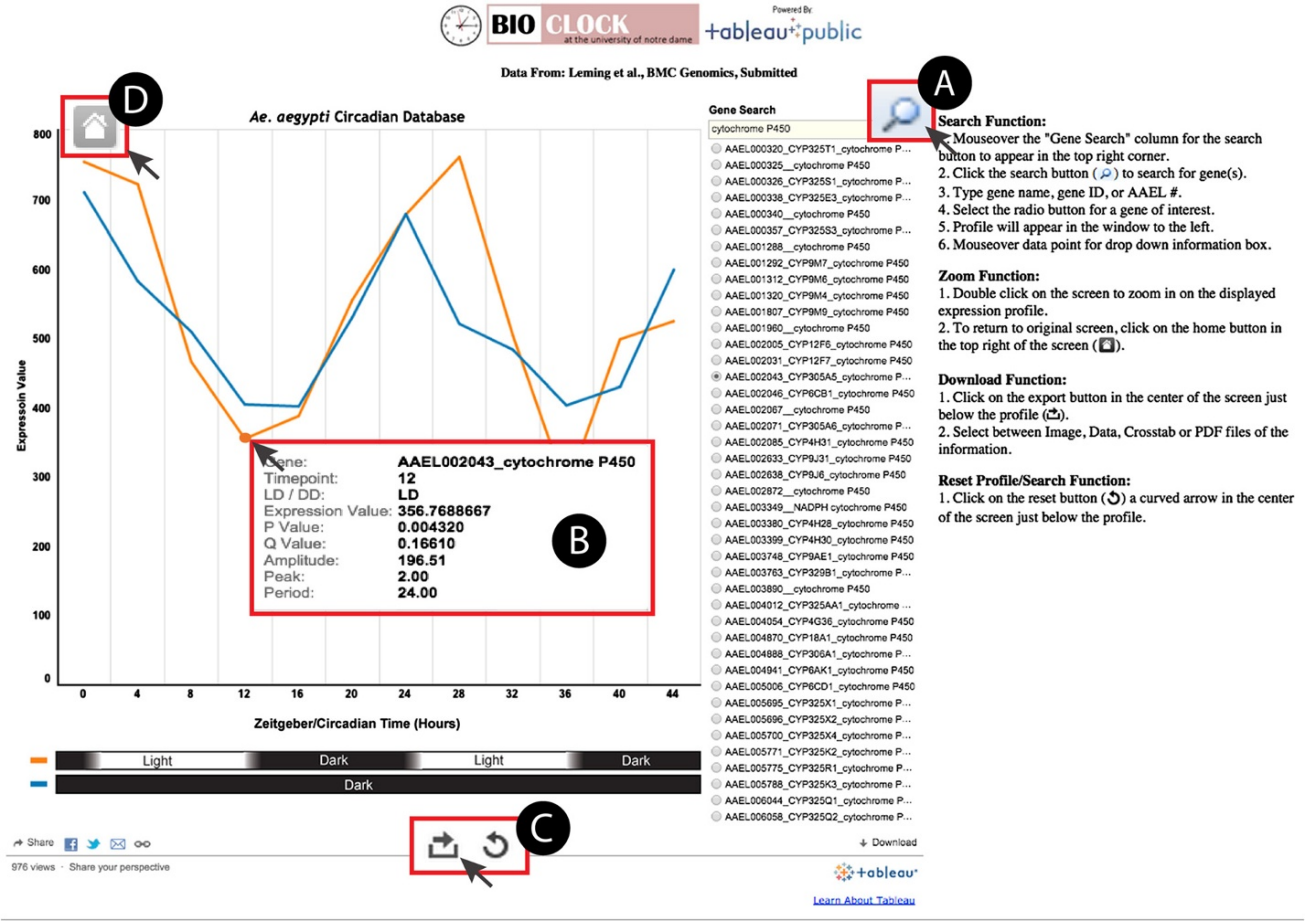
**Screenshot of the** ***Aedes aegypti*** **Circadian Database demonstrates the key features of the user interface.** In this example, tools are being utilized to identify the *period* gene (AAEL008141). **A**. On click, the search function allows for querying of gene names, gene descriptions and assigned AAEL numbers. **B**. The “mouseover” of each individual data point provides specific values for: (1) Timepoint, time (in hours) of collection of sample after onset of experiment (at ZT or CT 0), (2) LD/DD, light/dark or constant dark conditions of the sample, (3) Expression Value, raw fluorescence intensity of the data point, (4) Probability (*p*) value, for the individual gene that determines likelihood of rhythmicity (*p* <0.05 assigned as rhythmic), (5) False discovery rate (FDR) *q*-value for each gene (6) Amplitude, a measure of the maximum extent of oscillation of each optimal cyclical pattern, (7) Peak, time of day a gene was determined to have its highest intensity based on the cosine wave fit, and (8) Period, the time interval between one event in the cycle (e.g. peak or trough) in expression and the next sequential event in expression. The JTK_CYCLE algorithm was used to calculate the probability, amplitude, peak and period values. **C**. Image, Data, Crosstab, or PDF files can be generated and downloaded from the database. To download the data and visualizations for a given profile, click the download button depicted as a right facing arrow in an open box. Immediately to the right of the download button is a refresh button, identifiable as the circular left-facing arrow, that resets the database to its default settings. **D**. Double clicking on the profile screen allows the user to zoom in on a profile for close examination of profiles. Clicking the home button returns the screen to the original image.

The *Ae. aegypti* Circadian Database is hosted on Bioclock, and the interactive user interface is operated by Tableau Public. This platform was selected because it offered several potential advantages over the CircaDB platform. Specifically, Tableau Public possesses a user-friendly interface that provides interactive and downloadable visualizations with specification of actual expression values. The user interface is designed specifically for querying genes contained within this dataset. By selecting the “Gene Search” function the user can enter the VectorBase Gene ID, gene name or gene description to search for a specific gene or a group of genes (e.g. “cryptochrome P450”; Figure 4A). A list of genes matching that description is then populated. Upon selection, a temporal profile for the selected gene is generated in both LD and DD conditions giving the user information about rhythmicity under both diel and circadian regulation, respectively. Each profile gives the RMA normalized intensity values for all gene-specific probes on the chip at that time point. A cursor activated drop down box is generated on any individual point by “mouseover”. Information provided in this box includes the exact value of intensity of that point as well as the *p*-value of rhythmicity, the *q*-value for false discovery rates, estimated period length, estimated peak phase, and relative amplitude value as determined by JTK_CYCLE (Figure 4B). Furthermore, this database allows for the data and visualizations of each profile to be downloadable as Image, Data, Crosstab, or PDF files (Figure 4C). Finally, each profile can be zoomed in on for inspection of low amplitude rhythms (Figure 4D).

Through the course of generating this database we were able to estimate the total percentage of rhythmic/circadian genes in *Ae. aegypti* heads. Based on our *p* < 0.05 significance level, of the total 15,202 genes represented on the chip, 1,035 (6.8%) were rhythmic under LD conditions, and 887 (5.8%) were observed as rhythmic under DD and can therefore be considered to be under circadian clock regulation. When we excluded genes with average expression below background levels and only considered genes called present in the head, we identified 13,108 and 13,145 genes remaining in LD and DD, respectively. Therefore our estimates for rhythmic genes as a factor of genes expressed in the head were determined to be 7.9% and 6.7% for LD and DD, respectively.

## Conclusions

This work documents the experimental strategies used to observe the role of the light/dark cycle and the circadian clock on the expression of individual genes in the disease vector *Ae. aeygypti*. The *Ae. aegypti* Circadian Database was designed as a community resource, allowing vector biology and chronobiology researchers to access this information in a straightforward manner.

## Availability and requirements

The database is publicly available at http://www.nd.edu/~bioclock/. The database and associated files are open source and have been deposited on NCBI-GEO [GEO accession# GSE60496] and VectorBase.org.

## Abbreviations

BLAST: Basic local alignment search tool
CT: Circadian time
DD: Dark/Dark
GEO: Gene expression omnibus
LD: Light/Dark
NCBI: National Center for Biotechnology Information
TTFL: Transcriptional-translational feedback loop
ZT: Zeitgeber time.

## References

[1] Guzman MG, Halstead SB, Artsob H, Buchy P, Farrar J, Gubler DJ, Hunsperger E, Kroeger A, Margolis HS, Martinez E, Nathan MB, Pelegrino JL, Simmons C, Yoksan S, Peeling RW (2010). Dengue: a continuing global threat. Nat Rev Microbiol.

[2] Jentes ES, Poumerol G, Gershman MD, Hill DR, Lemarchand J, Lewis RF, Staples JE, Tomori O, Wilder-Smith A, Monath TP (2011). The revised global yellow fever risk map and recommendations for vaccination, 2010: consensus of the Informal WHO Working Group on Geographic Risk for Yellow Fever. Lancet Infect Dis.

[3] Staples JE, Breiman RF, Powers AM (2009). Chikungunya fever: an epidemiological review of a re-emerging infectious disease. Clin Infect Dis.

[4] Meireles-Filho AC, Kyriacou CP (2013). Circadian rhythms in insect disease vectors. Mem Inst Oswaldo Cruz.

[5] Allada R, Chung BY (2010). Circadian organization of behavior and physiology in *Drosophila*. Annu Rev Physiol.

[6] Dunlap J, Loros J, DeCoursey P (2004). Chronobiology: biological timekeeping.

[7] Lin Y, Han M, Shimada B, Wang L, Gibler TM, Amarakone A, Awad TA, Stormo GD, Van Gelder RN, Taghert PH (2002). Influence of the period-dependent circadian clock on diurnal, circadian, and aperiodic gene expression in *Drosophila melanogaster*. Proc Natl Acad Sci U S A.

[8] Rund SS, Hou TY, Ward SM, Collins FH, Duffield GE (2011). Genome-wide profiling of diel and circadian gene expression in the malaria vector *Anopheles gambiae*. Proc Natl Acad Sci U S A.

[9] Ptitsyn AA, Reyes-Solis G, Saavedra-Rodriguez K, Betz J, Suchman EL, Carlson JO, Black WCT (2011). Rhythms and synchronization patterns in gene expression in the *Aedes aegypti* mosquito. BMC Genomics.

[10] Yuan Q, Metterville D, Briscoe AD, Reppert SM (2007). Insect cryptochromes: gene duplication and loss define diverse ways to construct insect circadian clocks. Mol Biol Evol.

[11] Ozturk N, Song SH, Selby CP, Sancar A (2008). Animal type 1 cryptochromes. Analysis of the redox state of the flavin cofactor by site-directed mutagenesis. J Biol Chem.

[12] Rubin EB, Shemesh Y, Cohen M, Elgavish S, Robertson HM, Bloch G (2006). Molecular and phylogenetic analyses reveal mammalian-like clockwork in the honey bee (*Apis mellifera*) and shed new light on the molecular evolution of the circadian clock. Genome Res.

[13] Gentile C, Meireles-Filho AC, Britto C, Lima JB, Valle D, Peixoto AA (2006). Cloning and daily expression of the timeless gene in *Aedes aegypti* (Diptera:Culicidae). Insect Biochem Mol Biol.

[14] Gentile C, Rivas GB, Meireles-Filho AC, Lima JB, Peixoto AA (2009). Circadian expression of clock genes in two mosquito disease vectors: cry2 is different. J Biol Rhythms.

[15] Duffield GE (2003). DNA microarray analyses of circadian timing: the genomic basis of biological time. J Neuroendocrinol.

[16] Kojima S, Shingle DL, Green CB (2011). Post-transcriptional control of circadian rhythms. J Cell Sci.

[17] Ceriani MF, Hogenesch JB, Yanovsky M, Panda S, Straume M, Kay SA (2002). Genome-wide expression analysis in *Drosophila* reveals genes controlling circadian behavior. J Neurosci.

[18] Wijnen H, Naef F, Boothroyd C, Claridge-Chang A, Young MW (2006). Control of daily transcript oscillations in *Drosophila* by light and the circadian clock. PLoS Genet.

[19] Rund SS, Gentile JE, Duffield GE (2013). Extensive circadian and light regulation of the transcriptome in the malaria mosquito *Anopheles gambiae*. BMC Genomics.

[20] Rund SS, Bonar NA, Champion MM, Ghazi JP, Houk CM, Leming MT, Syed Z, Duffield GE (2013). Daily rhythms in antennal protein and olfactory sensitivity in the malaria mosquito *Anopheles gambiae*. Sci Rep.

[21] Balmert NJ, Rund SS, Ghazi JP, Zhou P, Duffield GE (2014). Time-of-day specific changes in metabolic detoxification and insecticide resistance in the malaria mosquito *Anopheles gambiae*. J Insect Physiol.

[22] Boorman J (1961). Observations on the habits of mosquitoes of Plateau Province, Northern Nigeria, with special reference to *Aedes vittatus* (Bigot). Bull Ent Res.

[23] McClelland G (1959). Observations on the mosquito, *Aedes (Stegomyia) aegypti (L.)*, in East Africa: I - the biting cycle in an outdoor population at Entebbe, Uganda. Bull Ent Res.

[24] Haddow A, Gillett JD (1957). Observations on the oviposition-cycle of *Aedes (Stegomyia) aegypti (Linnaeus)*. Ann Trop Med Parisit.

[25] Gillett J, Haddow A, Corbet P (1962). The sugar-feeding cycle in a cage-population of mosquitoes. Entomol Exp Appl.

[26] Clements A (1999). The biology of mosquitoes.

[27] Yang YY, Liu Y, Teng HJ, Sauman I, Sehnal F, Lee HJ (2010). Circadian control of permethrin-resistance in the mosquito *Aedes aegypti*. J Insect Physiol.

[28] Hu X, Leming MT, Metoxen AJ, Whaley MA, O'Tousa JE (2012). Light-mediated control of rhodopsin movement in mosquito photoreceptors. J Neurosci.

[29] Hughes ME, Hogenesch JB, Kornacker K (2010). JTK_CYCLE: an efficient nonparametric algorithm for detecting rhythmic components in genome-scale data sets. J Biol Rhythms.

[30] Wendell MD, Wilson TG, Higgs S, Black WC (2000). Chemical and gamma-ray mutagenesis of the white gene in *Aedes aegypti*. Insect Mol Biol.

[31] Rund SS, Lee SJ, Bush BR, Duffield GE (2012). Strain- and sex-specific differences in daily flight activity and the circadian clock of *Anopheles gambiae* mosquitoes. J Insect Physiol.

[32] Tomchaney M, Mysore K, Sun L, Li P, Emrich SJ, Severson DW, Duman-Scheel M (2014). Examination of the genetic basis for sexual dimorphism in the *Aedes aegypti* (dengue vector mosquito) pupal brain. Biol Sex Differ.

[33] Irizarry RA, Bolstad BM, Collin F, Cope LM, Hobbs B, Speed TP (2003). Summaries of Affymetrix GeneChip probe level data. Nucleic Acids Res.

[34] Irizarry RA, Hobbs B, Collin F, Beazer-Barclay YD, Antonellis KJ, Scherf U, Speed TP (2003). Exploration, normalization, and summaries of high density oligonucleotide array probe level data. Biostatistics.

[35] de Hoon MJ, Imoto S, Nolan J, Miyano S (2004). Open source clustering software. Bioinformatics.

[36] Chauhan C, Behura SK, Debruyn B, Lovin DD, Harker BW, Gomez-Machorro C, Mori A, Romero-Severson J, Severson DW (2012). Comparative expression profiles of midgut genes in dengue virus refractory and susceptible *Aedes aegypti* across critical period for virus infection. PLoS One.

[37] Eisen MB, Spellman PT, Brown PO, Botstein D (1998). Cluster analysis and display of genome-wide expression patterns. Proc Natl Acad Sci U S A.

[38] Saldanha AJ (2004). Java Treeview–extensible visualization of microarray data. Bioinformatics.

[39] Morlais I, Severson DW (2001). Identification of a polymorphic mucin-like gene expressed in the midgut of the mosquito, *Aedes aegypti*, using an integrated bulked segregant and differential display analysis. Genetics.

[40] Panda S, Antoch MP, Miller BH, Su AI, Schook AB, Straume M, Schultz PG, Kay SA, Takahashi JS, Hogenesch JB (2002). Coordinated transcription of key pathways in the mouse by the circadian clock. Cell.

[41] Pizarro A, Hayer K, Lahens NF, Hogenesch JB (2013). CircaDB: a database of mammalian circadian gene expression profiles. Nucleic Acids Res.

[42] Behura SK, Gomez-Machorro C, Harker BW, de Bruyn B, Lovin DD, Hemme RR, Mori A, Romero-Severson J, Severson DW (2011). Global cross-talk of genes of the mosquito *Aedes aegypti* in response to dengue virus infection. PLoS Negl Trop Dis.

